# Cause or effect? Interpreting emerging evidence for dysbiosis in systemic sclerosis

**DOI:** 10.1186/s13075-019-1872-4

**Published:** 2019-03-27

**Authors:** Christopher P. Denton, Charles Murray

**Affiliations:** 1 Centre for Rheumatology and Connective Tissue Diseases, Division of Medicine, University College London, Rowland Hill Street, London, NW3 2PF UK; 2 Royal Free London NHS Foundation Trust, Pond Street, London, NW3 2QG UK

## Abstract

Systemic sclerosis (SSc) is an immune-mediated fibrotic disease affecting skin, lung and gut which are all sites with an established microbiome. Altered microbial flora may occur and contribute to the initiation, progression or severity of disease. However, dysbiosis could also be secondary to the disease or immunosuppressive therapy. Here we consider how lessons could be learned from gastroenterology, a speciality where dysbiosis is strongly implicated in disease mechanism and treatment. This could be highly relevant to molecular pathology of skin in SSc and could drive the inflammatory gene signature observed in some skin biopsies.

Modern molecular techniques have allowed unprecedented interrogation of genomics, and this approach offers insights into microbial colonisation and speciation in the skin and other organs. This approach has been used by Johnson et al. [1] to explore the possible role of skin dysbiosis in systemic sclerosis (SSc). This follows a well-trodden route of investigation that is perhaps best delineated for the microbiome of the gastrointestinal tract. The interface between susceptibility and environment is likely to be especially relevant in uncommon acquired diseases, and a striking feature of systemic sclerosis is early and important involvement of the skin, gut and respiratory system. It is noteworthy that these all represent locations where there is a well-established microbial flora and sites for immune surveillance and immunoregulation that could be altered by changes in the microbiome.

In the skin, there are surprising findings in this report. Unexpected species are present, and this might indicate factors that trigger, amplify or promote progression of skin involvement. In addition, there is a clear change in the molecular microbial signature with decreased lipophilic taxa and a marked increase in a wide range of gram-negative taxa. It is notable that there is not a clear observed association with more severe diffuse forms of SSc which would have perhaps supported a role in progression of severity. The possible link with inflammatory gene expression signatures in the skin raises the possibility that microbial environment might be stimulating elements of the innate or adaptive immune system relevant to pathogenesis. However, the skin, gut and lung are also very much altered structurally and architecturally in SSc, and so these findings might reflect that perhaps the skin in SSc is especially susceptible to colonisation by these microorganisms and reflect an altered host rather than a role in aetiopathogenesis. In addition, the pharmacological and non-drug therapies offered to all SSc cases might impact on gut flora. Antibiotics are used regularly to treat secondary infection and in some cases are administered for prolonged periods.

If the significance of dysbiosis is to be proven, it is probably helpful to look at studies within the gastrointestinal tract. Although relatively sparse data are available for SSc [2, 3], there is a large literature related to other forms of GI disease. In multiple GI diseases including irritable bowel syndrome [4], inflammatory bowel disease [5] and liver disease [6], the putative, causative or associated role of the gut microbiome is still poorly understood, and more widely the association with gut microbiota diversity and health has led to a rapidly evolving understanding of the wide effects of diet and disease. In fact, the implications of diet on gut microbiota can be profound [7], and since poor microbiota diversity has been demonstrated to be associated with poor health outcomes [8], the gut microbiota appear central in many disease processes, whether predominantly due to gut-related illness or indeed in association with both physical and mental health outcomes.

The findings in this and other studies raise intriguing questions about cause and effect in SSc. Gut microbiota is affected markedly by changes in diet, and dysbiosis has been associated with gastrointestinal features in SSc [3]. Whether this dysbiosis is associated with changes in gut motility and subsequent fermentation for example, or even secondary changes in dietary preferences, or whether it is a driver in aetiopathogenesis of SSc gut disease is not clear. In a mouse model of scleroderma associated with anti-topoisomerase-I immunity, for example, skin and lung fibrosis were exacerbated by perinatal antibiotic use [8]. Could therefore scleroderma be a disease primarily driven from the gut by perturbations in the gut microbiome at an early age? There are suggestions in other immune diseases such as inflammatory bowel disease that this may be the case [9].

In conclusion, the present study of skin microbiome is small and requires further confirmation and study. However, it is likely that complex diseases such as SSc will inevitably be determined in part by factors related to the microbiome and that this may offer important potential for symptomatic or even disease-modifying treatments and could offer insight into the benefits reported by patients using interventions predicted to impact on skin, lung or gut dysbiosis.

## Abbreviations

SSc: Systemic sclerosis
